# Investigation of serum levels of orexin‐A, transforming growth factor β, and leptin in patients with multiple sclerosis

**DOI:** 10.1002/jcla.24170

**Published:** 2021-12-11

**Authors:** Sepideh Moharami, Alireza Nourazarian, Masoud Nikanfar, Delara Laghousi, Behrouz Shademan, Omid Joodi Khanghah, Fatemeh Khaki‐Khatibi

**Affiliations:** ^1^ Neurosciences Research Center (NSRC) Tabriz University of Medical Sciences Tabriz Iran; ^2^ Department of Biochemistry and Clinical Laboratories Faculty of Medicine Tabriz University of Medical Sciences Tabriz Iran; ^3^ Department of Basic Medical Sciences Khoy University of Medical Sciences Khoy Iran; ^4^ Department of Neurology Faculty of Medicine Tabriz University of Medical Sciences Tabriz Iran; ^5^ Social Determinant of Health Research Center Health Management and Safety Promotion Research Institute Tabriz University of Medical Sciences Tabriz Iran; ^6^ Department of Medical Biology Faculty of Medicine EGE University Izmir Turkey

**Keywords:** Body Mass Index, leptin, multiple sclerosis, orexin‐A, TGF‐β

## Abstract

**Background:**

Multiple sclerosis (MS) is a chronic inflammatory and autoimmune disease affecting various inflammatory and nutritional parameters. Therefore, this study aimed to investigate the relationship between the Body Mass Index (BMI) of MS patients and the serum levels of leptin, orexin‐A, and Transforming Growth Factor β (TGF‐β).

**Methods:**

This cross‐sectional study included 25 patients suffering from MS and 40 healthy individuals as the case and control groups, respectively. The serum levels of leptin, orexin‐A, and TGF‐β were assessed in the participants using the Enzyme‐Linked Immunosorbent Assay methods. Moreover, data were analyzed using the descriptive statistical indices, *t*‐test, chi‐square test, and linear regression test.

**Results:**

According to our results, the participants’ mean age was 38.04 ± 7.53 and 40.23 ± 5.88 in the case and control groups, respectively. Also, the groups were not significantly different in gender, age, alcohol consumption, and smoking (*p* > 0.05). It was found that the mean serum levels of orexin‐A and TGF‐β were significantly lower in the MS patients compared to the control group, while the mean serum leptin levels were significantly higher (42.8 vs. 18.9 ng/ml, *p* < 0.001). Moreover, there was no significant relationship between the BMI of the MS patients and their serum levels of orexin‐A, TGF‐β, and leptin (*p* > 0.05).

**Conclusions:**

In conclusion, we found significantly lower levels of orexin‐A and TGF‐β and a significantly higher level of leptin in the MS patients compared to the control group. In addition, there was no significant relationship between the BMI and the serum levels of orexin‐A, TGF‐β, and leptin in MS patients.

## Graphical abstract

Background: Multiple Sclerosis (MS) is a chronic inflammatory and autoimmune disease related to various inflammatory and nutritional parameters. This study was conducted to determine the relationship between serum leptin, orexin‐A, and TGF‐β levels and BMI in MS patients. Methods: This cross‐sectional study included 25 patients with relapsing‐remitting multiple sclerosis (RRMS) and 40 healthy controls. Serum levels of leptin, orexin‐A and TGF‐β were measured by an enzyme‐linked immunosorbent assay (ELISA). Data was analyzed using descriptive statistics, t‐tests, chi‐square tests, and linear regression tests. 65 volunteers, including 25 MS patients and 40 healthy individuals, were enrolled in the study. Results: The mean ages of subjects in the case and control groups were 38.04 ± 7.53 and 40.23 ± 5.88, respectively. There were no statistically significant differences between the case and control groups in sex, age, alcohol consumption, and cigarette consumption (*p* > 0.05). Mean serum levels of orexin‐A and TGF‐ss were lower in patients with MS than in healthy controls, but leptin was higher (42.8 vs. 18.9 ng/ml, *p* < 0.001). The relationship between BMI and serum levels of orexin‐A, TGF‐ss, and leptin in MS patients was not statistically significant (*p* > 0.05). Conclusion: Our results showed that serum levels of orexin‐A and TGF‐P were lower. The serum level of leptin was higher in patients with MS than in healthy controls. In addition, there was no statistically significant relationship between BMI and serum levels of orexin‐A, TGF‐ss, and leptin in patients with MS.

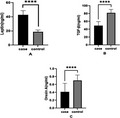

## 1 INTRODUCTION

Multiple sclerosis (MS) is a widespread and potentially devastating disease of the brain and spinal cord. This disease is widespread and affects more than 2.8 million people worldwide.^1^ It is also characterized by attacks by the immune system on a specific protective layer (myelin) surrounding neurons, resulting in sensory‐motor dysfunction. Ultimately, the disease may lead to degeneration or irreversible loss of the affected nerves.^2^ Despite the extensive research that has been conducted on MS, the exact etiology of the disease remains unclear.^3^, ^4^

It has been found that MS is associated with excessive T‐cell reactivity to various antigens and also with abnormal B‐cell responses.^1^, ^2^ Given the diversity of neurological impairments in different patients, the disease may manifest with different clinical presentations. The risk of developing MS is higher in individuals with a family history of autoimmune disease and those with specific risk factors for the disease, including obesity, Epstein‐Barr virus infection, vitamin D deficiency, smoking, and lack of sun exposure.^1^, ^4^ Several studies have found that obesity and high Body Mass Index (BMI) are associated with the development of various health problems and neurological diseases such as MS, Alzheimer's disease, and Parkinson's disease.^5^, ^6^

Obesity has been shown to play a critical role in MS pathogenesis. However, researchers are still investigating the processes by which obesity contributes to MS development. Several hypotheses have been proposed regarding the role of obesity in the development of MS. For example, one hypothesis is that obese individuals are more affected by vitamin D deficiency than normal‐weight individuals.^7^ In addition, some specific secretions of adipose tissue might contribute to the development of this disease.^8^ Orexin‐A, secreted by adipose tissue, is a neuropeptide that regulates various physiological functions such as sleep and eating behavior.^9^ This substance plays a crucial role in glucose and energy metabolism. Numerous studies have shown that orexin‐A has neuroprotective effects. Therefore, it may be beneficial for inflammatory neurological diseases such as MS.^8^, ^9^ Another potential compound is adiponectin, also known as leptin, which regulates eating behaviors in humans, such as craving, hunger, and suppression.^10^ In addition, leptin has pro‐inflammatory properties and functions. As an acute‐phase protein, its level is increased by inflammatory mediators and can influence the immune system by inducing the release of cytokines. An imbalance in leptin levels can impair immunity and exacerbate autoimmune diseases such as MS and related problems.^8^, ^9^ Another substance that may play a role in the relationship between obesity and the development of MS is Transforming Growth Factor β (TGF‐β). This substance has been shown to affect both the activity and growth of T helper cells and regulatory T cells, both of which are impaired in MS.^10^ Given the involvement of T helper cells in autoimmunity, the relationship between TGF‐β and neurodegenerative diseases such as MS, Alzheimer's disease, and Parkinson's disease has been extensively studied.^10^, ^11^, ^12^

In addition, BMI is a statistical measure of a person's weight and height. It is also an effective tool for assessing health status and can be used in studying the prevalence of obesity. This index indicates how well a particular person's height and weight match.^13^ As mentioned previously, leptin, TGF‐β, and orexins may play a role in the pathophysiology of MS. Therefore, this study aimed to investigate the serum concentrations of orexin‐A, TGF‐β, and leptin in MS patients and the relationship between these variables and BMI.

## 2 MATERIALS AND METHODS

### 2.1 Study population

This study was a cross‐sectional study approved by the ethics committee of Tabriz College of Medical Sciences with ethics code 1398.527. All study participants gave informed consent to participate. In addition, the objectives of the study were explained to them in detail. Participants included 25 patients with MS, 21 of whom had relapsing‐remitting MS (RRMS) and four had clinically isolated syndrome, and 40 healthy individuals as a control group. The case group included the patients who presented to Razi Hospital, Tabriz, Iran and were diagnosed by an experienced neuroscientist using standard biomarkers in serum and cerebrospinal fluid (CSF) for MS. In addition, patients underwent cerebral magnetic resonance imaging (MRI), and MRI images were evaluated using McDonald criteria. In addition, the absence of any gadolinium contrast enhancement was considered as no evidence of disease activity. Exclusion criteria also included alcohol consumption, smoking, cardiovascular disease, neurologic disease (not MS), pregnancy, and autoimmune diseases such as rheumatoid arthritis and lupus erythematosus. In addition, patients taking corticosteroids, serum lipid‐lowering drugs, and immunosuppressants were excluded.

### 2.2 Sample preparation

Participants were recruited over 6 months (from May 2020 to October 2020) from Razi Hospital, Tabriz, Iran. For the assessment of biochemical variables, each participant gave 10 ml of blood after fasting overnight. Samples were placed in tubes and centrifuged at 650 *g* for 4 min at 4°C. They were then stored at −80°C. All experiments were performed in the biochemical laboratory of the Pharmaceutical Research Center of Tabriz College of Medical Sciences.

### 2.3 Cholesterol and triglyceride assessments

Cholesterol levels were determined using the Pars Azmoon assay kit (Cat. No. 110 500 BT, Iran) and an automated biochemistry analyzer (BT3000). In this assay, quinone imine levels formed by cholesterol hydrolysis and oxidation are directly correlated with cholesterol levels and can be easily determined by spectrophotometry at a wavelength of 550 nm. In addition, the Pars Azmoon assay kit (Cat. No. 132 504 H917) and an automated biochemistry analyzer (Hitachi 917) were used to evaluate triglyceride levels. In this assay, quinone imine is generated by the reaction of glycerol with 4‐aminoantipyrine and phenol and can be determined by spectrophotometry at a wavelength of 550 nm.

### 2.4 Orexin‐A assessment

The Human Orexin‐A Enzyme‐Linked Immunosorbent Assay (ELISA) Kit (Cat. No. CSB‐E08859h from CUSBIO) was used to measure orexin‐A. This assay is based on the quantitative sandwich enzyme immunoassay technique. In this assay, a precoated antibody captures orexin‐A. After washing, a biotin‐conjugated antibody specific for orexin‐A is applied. Then the unbound antibody is removed, and avidin‐conjugated horseradish peroxidase (HRP) is added to the wells. After removing the unbound conjugated avidin, the resulting substrate is used to generate the color, and the optical density is measured at a wavelength of 450 nm.

### 2.5 TGF‐β assessment

TGF‐β level was determined using the Biosensis ELISA kit (Cat. No. BEK_2093_1P). In this assay, the captured antibody is immobilized in the wells, and the samples are placed in the wells. After washing, a biotinylated TGF‐β antibody is used as a detector. Then, the avidin‐biotin‐peroxidase complex that binds to the second antibody is added. The peroxidase substrate is used to generate a colored reaction product that can be measured at a wavelength of 450 nm.

### 2.6 Leptin assessment

According to the manufacturer's instructions, we used the ALPCO ELISA kit (Cat. No. 11‐LEPHU‐E01, USA). As with orexin‐A and TGF‐β, this assay is based on the sandwich enzyme immunoassay technique. Leptin in the samples is bound to the immobilized antibody and the biotinylated antibody to form a sandwich complex. After the addition of streptavidin‐HRP and substrate, the optical density of the stained product is measured at a wavelength of 450 nm.

### 2.7 BMI calculation

The weight and height of the participants were measured, and BMI values were calculated using the available standard tables and the following formula:

$$\text{BMI}\;\text{(kg}/m^{2})=\text{weight}\;\text{(kg)}/\text{squared}\;\text{height}\;\text{(m)}.$$

### 2.8 Statistical analysis

The categorical variables were described by frequency and percentage, whereas the mean and standard deviation (SD) were used for the continuous variables. In addition, the Kolmogorov‐Smirnov test was used to assess the normality of the distributions of the continuous variables. The *t*‐test and chi‐square test were used for comparisons between the groups of continuous and categorical variables. In addition, a logistic regression test was used to compare serum biomarker levels between groups. The relationship between BMI and biomarker levels in MS patients was also examined using the linear regression test. A *p* value of <0.05 was considered significant, and all data were analyzed using SPSS version 21 and Graph Pad Prism 8.

## 3 RESULTS

### 3.1 Demographics

This study included 65 participants, of whom 25 were MS patients and 40 were healthy controls. The demographic data of the participants are shown in Table 1. To our knowledge, the case and control groups did not differ significantly in terms of sex, age, alcohol consumption, and cigarette smoking (*p* > 0.05).

**TABLE 1:** Demographic and disease related characteristics of the study population

Variable	Total	Case	Control	*p*‐value*
Number	65	25	40	
Gender, *N* (%)				0.46
Male	33 (50.8%)	12 (48%)	21 (52.5%)	
Female	32 (49.2%)	13 (52%)	19 (47.5%)	
Mean age (SD)	39.38 (6.59)	38.04 (7.53)	40.23 (5.88)	0.224
OCP history, *N* (%)	–	–	–	–
Cigarette history, *N* (%)	3 (4.6%)	3(12%)	0	0.053
Alcohol use, *N* (%)	–	–	–	–
Diabetic disease, *N* (%)	2 (3.1%)	2 (8%)	0	0.14
Cancer history, *N* (%)	–	–	–	–
Duration of MS disease (year)		2.84 (1.8)	–	–

Abbreviation: SD, standard deviation.

* Chi‐squared test was used.

### 3.2 Biochemical and anthropometric characteristics

**FIGURE 1:**
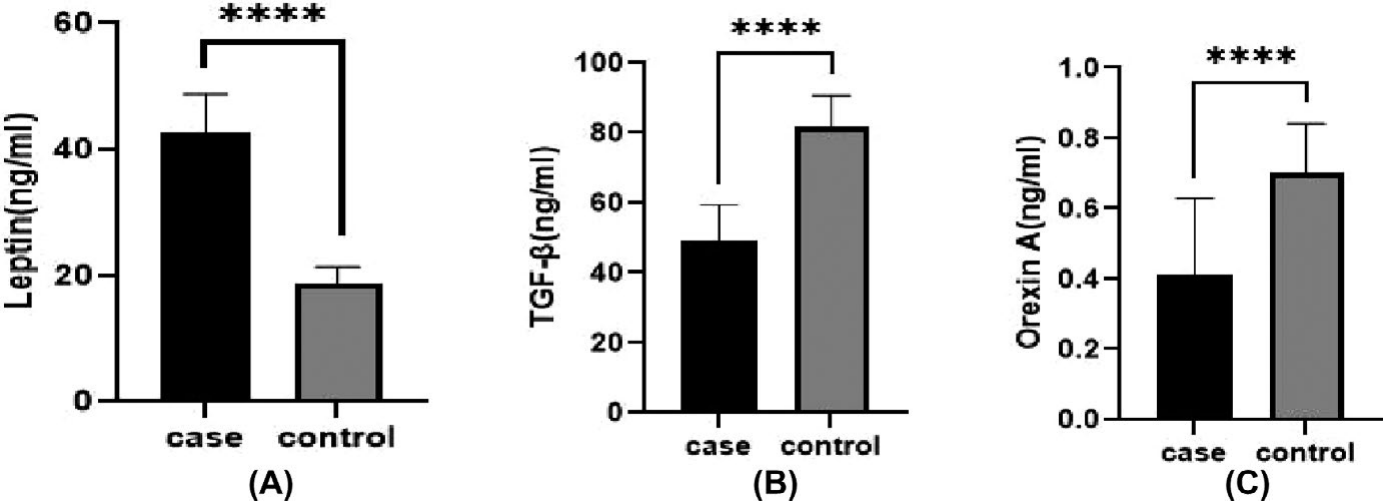
Comparison of serum levels of leptin (A), TGF‐β (B), and orexin‐A (C) in MS patients and the control group

As shown in Table 2, the study groups differed significantly in serum triglyceride and cholesterol levels (*p* < 0.0001). However, the groups did not differ significantly in anthropometric variables (*p* > 0.05). Furthermore, compared to healthy controls, serum levels of orexin‐A and TGF‐β were significantly lower in MS patients, whereas leptin levels were significantly higher (*p* 0.001, Figure 1).

**TABLE 2:** Biochemical and Anthropometric characteristics between cases and controls

Variable	Total	Patients with MS (*n* = 25)	Control groups (*n* = 40)	*p*‐value*
	Mean (SD)	Mean (SD)	Mean (SD)	
TG (mg/dl)	159.49 (77.5)	242 (64)	107.8 (15)	**<0.0001**
Chol (mg/dl)	155.92 (62.5)	228.4 (33)	110.6 (16.4)	**<0.0001**
Orexin‐A (ng/ml)	0.58 (0.22)	0.41 (0.21)	0.69 (0.14)	**<0.0001**
TGF‐β (ng/ml)	69.09 (18.1)	49.4 (10)	81.35 (8.7)	**<0.0001**
Leptin (ng/ml)	28.09 (12.4)	42.8 (5.9)	18.9 (2.8)	**<0.0001**
Weight	71.23 (13.9)	72.04(15)	70.73 (13.4)	0.502
Height	167.1 (10.5%)	166.4 (11)	167.5 (10.3)	0.640
BMI	25.4 (3.7)	25.8 (3.9)	25 (3.6)	0.490

Abbreviations: BMI, body mass index; Chol, Cholesterol; SD, standard deviation; TG, Triglyceride.

* *T*‐test was used.

### 3.3 Relationship between BMI and serum levels of orexin‐A, TGF‐β, and leptin in MS patients

According to the linear regression test results, serum levels of orexin‐A and TGF‐β increased by 0.2% and 24%, respectively, for each unit increase in BMI of patients from MS, whereas leptin levels decreased by 34%. However, the associations were not significant (*p* > 0.05; Table 3).

**TABLE 3:** The relationship between blood levels of orexin‐A, TGF‐β, leptin, and BMI in MS patients

	Unstandardized coefficients		Standardized coefficients	*t*	*p*‐value*
	*B*	Std. error	*r*		
Orexin‐A (ng/ml)	0.002	0.012	0.028	0.136	0.893
TGF‐B (ng/ml)	0.245	0.537	0.095	0.456	0.653
Leptin (ng/ml)	−0.340	0.310	−0.223	1.096	0.284

* Linear regression test was used.

## 4 DISCUSSION

As a neurodegenerative disease, MS causes inflammation in the central nervous system (CNS), leading to various manifestations of neuropathy.^13^ According to epidemiological studies, there is a significant association between the development of MS and diet, and the disease is more common in individuals with high fatty acid levels and vitamin D deficiency.^14^ As a result, serum levels of leptin, orexin‐A, and TGF‐β were measured in 25 MS patients in this study. In addition, the relationship between these variables and BMI was also evaluated in these patients.

According to our results, patients with MS had significantly higher leptin levels compared with healthy controls. In addition, triglyceride and cholesterol levels differed significantly between groups. Given the clinical and neurobiological complexity of the etiology and pathogenesis of MS, identifying biomarkers for the diagnosis of MS is challenging. The precise mechanisms of tissue and neuronal injury in this disease, including the relationships among inflammation, activated macrophages, axonal injury, and demyelination, have not been fully elucidated.^13^ It has been shown that MS patients on drug therapy have reduced serum and CSF levels of leptin. However, leptin levels are usually elevated during the acute phase of the disease.^14^, ^15^ Reduced leptin levels may ameliorate experimental autoimmune encephalomyelitis, an animal model of MS, by delaying disease onset and improving clinical symptoms.^15^

Leptin has been shown to influence innate and adaptive immunity and inflammatory responses by increasing monocyte proliferation, macrophage phagocytic activity, and production of pro‐inflammatory cytokines such as interleukin 6 (IL‐6) and IL‐12. In acquired immunity, leptin promotes naive T cell proliferation, T helper 1 (Th1) differentiation, and the production of tumor necrosis factor by memory T cells. In addition, it induces the proliferation of pro‐inflammatory Th1 cells, which contribute to the production of pro‐inflammatory cytokines such as interferon (INF) and IL‐2. In addition, leptin suppresses the production of Th2 cytokines such as IL‐4 and IL‐10, resulting in a pro‐inflammatory immune response.^16^

As mentioned previously, the MS patients in this study had significantly higher serum and CSF levels of leptin than healthy controls, suggesting that leptin may contribute to disease severity and subsequent disability.^17^ Based on this data, MAG may play a role in MS.

As a cytokine produced by monocytes, smooth muscle cells, and endothelial cells, TGF‐β is involved in tissue growth, extracellular matrix synthesis, and immunomodulation by inhibiting inflammatory responses and stimulating the differentiation of regulatory T cells.^16^ Mice lacking TGF‐β receptors have been shown to develop the inflammatory disease. The inability of T cells in TGF‐β secretion contributes to the development of autoimmune diseases.^17^ In addition, TGF‐β is associated with an increase in the levels of several cytokines, including IL‐2, IL‐6, and IL‐12.^18^ Therefore, regulation of TGF‐β levels can be used as a therapeutic approach. According to one study, TGF‐β levels are reduced in MS patients and are associated with increased IL‐2 and IL‐6 levels.^16^ Another study reported a significant decrease in TGF‐β expression in patients with RRMS, consistent with our findings.^17^ In this study, TGF‐β levels were lower in MS patients compared with the control group, which is consistent with previous studies.

Orexin‐A has been shown to increase appetite while decreasing metabolism.^16^, ^17^ According to our results, orexin‐A levels differed significantly between the MS patients and healthy controls. Some diseases affecting the CNS, such as MS, may decrease the release of this neuropeptide. Unfortunately, the importance of nutritional status is often underestimated by patients and physicians. The imbalance of the levels of orexin‐A and leptin likely affects the eating behavior of patients. However, the nutritional recommendations required for MS patients are usually controversial and have not been extensively studied.^18^ According to studies, orexins have neuroprotective and immunoregulatory (i.e., anti‐inflammatory) properties. Therefore, they may have therapeutic potential in various pathologies with a single immune component, including MS, Alzheimer's disease, obesity, septic shock, and cancer.^19^

Our results showed no significant association between BMI and serum levels of orexin‐A, TGF‐β, and leptin. With the occurrence of MS, obesity may exacerbate the disease and affect the response to treatment, and therefore, it may be a significant risk factor for the disease.^20^ However, the exact relationship between increased BMI and MS is not well studied, while some studies have found a direct association between BMI and disability in RRMS patients. One study reported that women with higher BMI were more likely to develop MS earlier in adulthood, which could be prevented by appropriate lifestyle and dietary habits.^21^ In addition, high levels of pro‐inflammatory cytokines and leptin and low levels of anti‐inflammatory cytokines were observed in obese patients with MS.^22^ Interestingly, we found no correlation in BMI between the case and control groups. Although the patients with MS had a good appetite, significantly higher leptin levels, and significantly lower orexin‐A levels than the control group, BMI did not differ between the groups, consistent with some other studies.^23^, ^24^ How can this contradictory finding be explained? Studies have reported the development of leptin resistance in individuals consuming a high‐fat diet.^25^, ^26^ As shown in Table 2, patients with MS had higher triglyceride and cholesterol levels compared with healthy participants, which may have led to leptin resistance in these patients.

This study aimed to investigate the relationship between MS and obesity. In addition, we attempted to illustrate the mechanism by which obesity may influence the development of MS, although we did not find a significant relationship between the two. However, this observation can be explained by the small sample size in this study, which was much more important in other studies investigating the association between obesity and MS.^27^ This small sample size was due to our limitations, including assessing three biochemical parameters and having insufficient participants. In addition, we used extensive exclusion criteria to exclude potential therapies. Therefore, many patients were excluded during the study. Larger sample size could lead to better statistical power and confirm our results.

## 5 CONCLUSION

According to the linear regression test results, serum levels of orexin‐A and TGF‐β increased by 0.2% and 24%, respectively, for each unit increase in BMI of patients from MS, whereas leptin levels decreased by 34%. However, the correlations were not significant. A high‐fat diet may lead to leptin resistance. In this study, the MS patients had higher triglyceride and cholesterol levels compared with healthy participants, leading to leptin resistance in these patients. Therefore, further studies are needed to clarify these ambiguities. In addition, our results showed that serum levels of leptin were significantly higher, whereas serum levels of TGF‐β and orexin‐A were significantly lower in patients with RRMS. These changes can be used for early detection of MS and evaluation of treatment progression. However, further studies are needed for definitive confirmation.

## CONFLICT OF INTEREST

The authors declare there are no conflicts of interest.

## AUTHOR CONTRIBUTIONS

S M: Writing—original draft. A N: Conceptualization, Writing—original draft. M N: Data curation. D L: Methodology. B S: Methodology, Data curation. O J k: Methodology. F k: Conceptualization, Methodology, Writing—review & editing.

## Supporting information

Supinfo S1

## Data Availability

The data and materials used in this study are available.
