# Supplementary figures and images for: Investigation of serum levels of orexin‐A, transforming growth factor β, and leptin in patients with multiple sclerosis
Source: J Clin Lab Anal. 2021 Dec 11;36(1):e24170. doi: 10.1002/jcla.24170 (PMC8761413; doi:10.1002/jcla.24170)

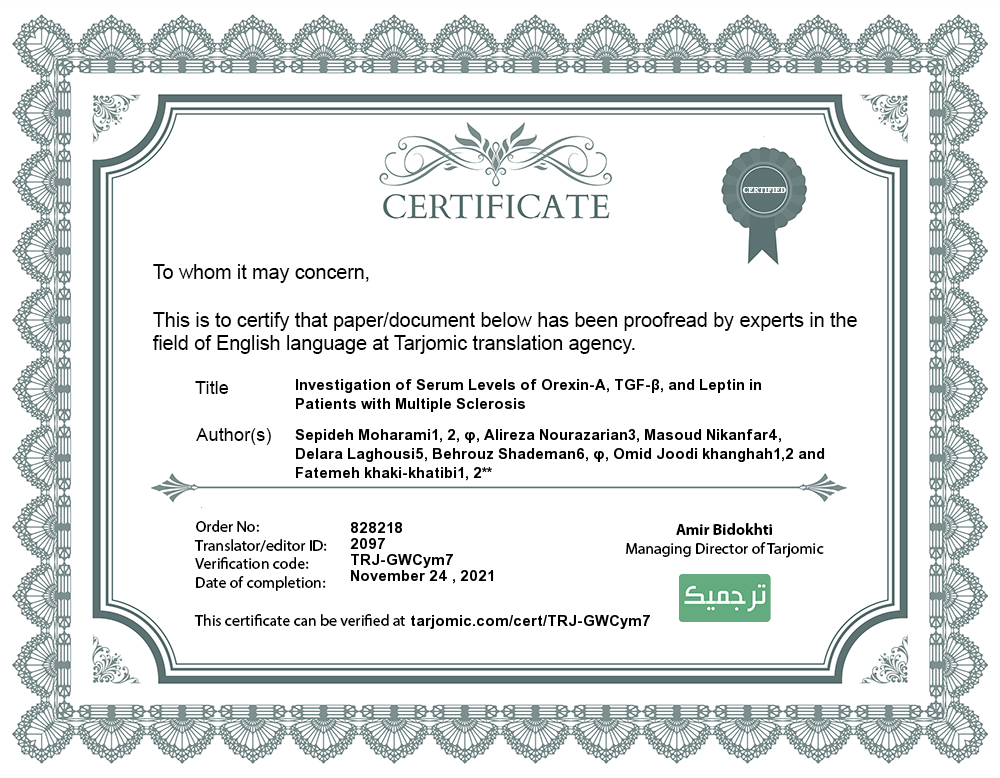

Supplement: Supplementary file 1 — Supinfo S1 [file JCLA-36-e24170-s001.png]
